# Proteomic signatures identify significantly different inflammatory breast tissue microenvironments depending on mammographic density or estrogen exposure

**DOI:** 10.1038/s41523-026-01046-4

**Published:** 2026-09-04

**Authors:** Menikae K. Heenkenda, Annelie Abrahamsson, Peter Lundberg, Charlotta Dabrosin

**Affiliations:** 1 Department of Biomedical and Clinical Sciences, Linköping University, Linköping, Sweden; 2 Clinical Department of Oncology, Region Östergötland, Linköping, Sweden; 3 Clinical Department of Medical Radiation Physics, Region Östergötland, Linköping, Sweden; 4 Center for Medical Image Science and Visualization (CMIV), Linköping University, Linköping, Sweden; 5 Department of Health, Medicine, and Caring Sciences, Linköping University, Linköping, Sweden; 6 Clinical Department of Radiology, Region Östergötland, Linköping, Sweden

**Keywords:** Biomarkers, Cancer, Cell biology, Oncology

## Abstract

Breast density and estrogen exposure are two major risk factors for breast cancer; however, the underlying biological mechanisms remain incompletely understood. Here, we investigated the extracellular proteomic landscape of normal human breast tissue in situ to define the microenvironment associated with these risk factors. Forty-two postmenopausal women with nondense or dense breasts and 19 premenopausal women underwent microdialysis. We quantified 461 inflammatory proteins in breast tissue and matched subcutaneous fat, enabling discrimination between local and systemic alterations. Breast density was assessed using magnetic resonance imaging. Dense breast tissue exhibited a distinct protein signature characterized by immune-related signaling, altered lipid metabolism, and the extracellular presence of intracellular proteins, consistent with cellular stress and immune modulation, with limited changes in angiogenic and extracellular matrix remodeling proteins. In contrast, estrogen-exposed breasts displayed a proteomic profile dominated by pro-inflammatory cytokines, angiogenic factors, extracellular matrix remodeling proteins, and complement activation, indicative of a dynamic and pro-tumorigenic microenvironment. These findings demonstrate that breast density and estrogen exposure are associated with fundamentally distinct breast microenvironments, both permissive for tumor progression. These breast-specific protein signatures provide mechanistic insight into how these risk factors contribute to cancer development and suggest that effective prevention strategies may require differential targeting.

## Introduction

Inflammation, one of the hallmarks of cancer, shapes the tissue microenvironment and may exert both tumor-promoting and tumor-suppressive effects^1^. The role of inflammation in tumor growth and metastasis has been demonstrated across numerous cancer types, including breast cancer^2,3^. Inflammatory bioactive molecules are released into the interstitial fluid of the microenvironment, where they influence angiogenesis, reorganization of the extracellular matrix (ECM), epithelial–mesenchymal transition (EMT), cancer cell proliferation, and disease progression^4–6^. Whether aberrant inflammation in the breast microenvironment is linked to established risk factors for breast cancer remains to be determined.

Breast cancer is the most common malignancy among women in the Western world, and its incidence continues to rise^7^. Although major advances in breast cancer therapy over recent decades have substantially improved survival, disease prevention remains the most effective strategy to reduce mortality and morbidity. However, significant gaps remain in our understanding of normal human breast pathophysiology, which is essential for the development of preventive interventions. More than 90% of breast cancers are sporadic, *i.e*. occurring in the absence of known genetic mutations. The two major independent risk factors for sporadic breast cancer are exposure to sex steroids and high mammographic breast density^8,9^. To date, the biological mechanisms underlying these two risk factors remain poorly understood.

Exposure to endogenous and exogenous sex steroids, including estrogens, is a well-established risk factor for breast cancer^8^. Sex steroids play a critical role in regulating breast epithelial cells as well as epithelial–stromal interactions within the breast microenvironment^10–16^. The anti-estrogen tamoxifen may theoretically be used as preventive therapy, but its use is limited by significant side effects, leading to treatment discontinuation in up to 50% of women with breast cancer^17^.

Women with dense breasts have a 4–6-fold increased risk of breast cancer compared with women with nondense breasts, and it has been estimated that women with more than 50% dense breast area account for approximately 30% of all breast cancer cases^9^. In normal breast tissue, epithelial cells constitute only 5–10% of the total tissue volume, and there are no conclusive data demonstrating differences in the quantity or activity of epithelial cells in relation to breast density^18–20^. Instead, the primary distinction between dense and nondense breast tissue lies in the stromal composition: dense breasts contain abundant collagen-rich stroma, whereas nondense breasts are predominantly composed of adipose tissue^19^. No association between circulating estrogen levels and breast density has been detected^9^.

For both major risk factors, the breast microenvironment, rather than the epithelial compartment, appears to play a central role in determining breast cancer risk, as stromal activation is a prerequisite for invasive cancer development.

In this study, we used microdialysis to sample extracellular proteins directly from live breast tissue in postmenopausal women with either dense or nondense breasts, as determined by mammography, as well as in premenopausal women. To distinguish breast-specific alterations from systemic effects, microdialysis was also performed in abdominal subcutaneous fat in all participants. Proteomic profiling of the collected microdialysates was conducted, and 461 inflammatory proteins were quantified using proximity extension assay (PEA).

We identified two distinct breast tissue–specific protein profiles associated with either breast density or local breast estradiol levels. In estrogen-exposed breast tissue, pathways related to inflammation, angiogenesis, ECM remodeling, and complement activation were predominantly upregulated. In contrast, the protein profile associated with dense breast tissue suggested immune evasion, altered lipid metabolism, and the extracellular presence of intracellular proteins indicative of cellular stress responses and immune surveillance.

Taken together, these findings indicate that preventive strategies targeting these established breast cancer risk factors must be tailored, as the underlying molecular landscapes differ substantially between estrogen-driven and density-associated risk profiles.

## Results

### Characterization of the subjects

There were no significant differences in body mass index (BMI), age, reproductive factors, previous hormonal use, or use of other medications or medical history between the postmenopausal women with dense or nondense breasts. Demographic and clinical data are presented in Table 1. Local breast estradiol levels were (pmol/L mean ± SD) 40 ± 14 vs 40 ± 12 in the dense and nondense groups, respectively.

**Table 1:** Characteristics of the included postmenopausal women with either nondense (BI-RADS A) or dense (BI-RADS D) breast tissue as depicted on mammography

	Dense *n* = 20	Nondense *n* = 20	*P*-value
Age (years)	64 ± 5.8	65 ± 5.2	0.2
Body Mass Index (kg/m^2^)	24 ± 3.3	25 ± 3.2	0.2
Menarche	13 ± 1.5	13 ± 1.6	0.7
Parity	1.8 ± 1.2	2.1 ± 1.3	0.3
Years since menopause	14 ± 5.7	14 ± 5.9	0.9
Prior hormonal contraceptives	17	17	1
Ongoing HRT	0	0	1
Prior HRT	6	8	0.7
Years since stopping HRT	10.7 ± 3	11 ± 1.4	0.6
Ongoing vaginal estrogen	5	6	1
Antihypertensives/Statins	6	9	0.7
SSRI	3	0	0.2

Mean ± SD. HRT.

(hormonal replacement therapy), SSRI (selective serotonin reuptake inhibitor).

LTF of breast tissue in women with dense breasts was 46 ± 23% vs. 3 ± 2.5% in nondense breasts.

BMI of the premenopausal women was 24 ± 1.5 (mean ± SD) and local breast estradiol levels were 196 ± 40 pmol/L (mean ± SD). None of the premenopausal women had any ongoing medication.

Premenopausal women have dense breast on mammography per se. To avoid any skewing of the results when comparing pre- and postmenopausal breast tissue, only the postmenopausal group with dense breast tissue was included in these analyses.

### Different expression profiles of protein in dense or estrogen exposed breast tissue

**Fig. 1:**
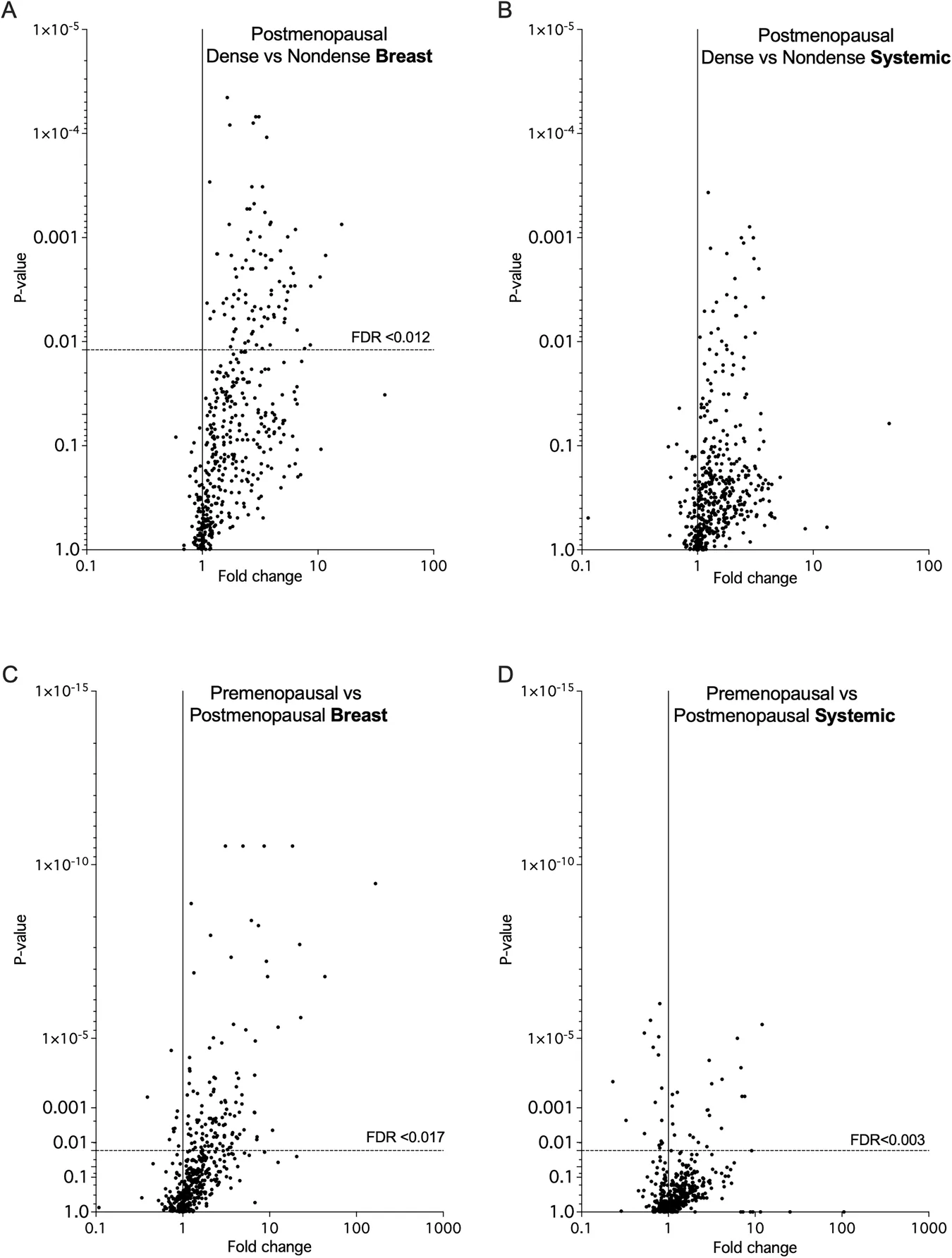
Different extracellular proteins signatures in vivo in dense or estrogen exposed normal human breast tissue. Microdialysis was performed in breast tissue of healthy postmenopausal women with dense, *n* = 20, or nondense, *n* = 20, breast and healthy premenopausal women, *n* = 19. One catheter was inserted in breast tissue and another in abdominal subcutaneous fat as a proxy for systemic alterations. 461 individual proteins in the microdialysates were quantified using proximity extension assay. **A** Proteins in dense vs nondense normal breast tissue from postmenopausal women. **B** Proteins in abdominal s.c. fat from postmenopausal women with dense or nondense breasts. **C** Proteins in premenopausal (high estradiol levels) vs postmenopausal (low estradiol levels) dense breast tissue. **D** Proteins in abdominal s.c. fat from the same women as in **C**. FDR, False Discovery Rate.

After FDR correction, *P* < 0.012, a total number of 93 proteins were significantly changed in dense breast tissue vs. nondense breasts, Fig. 1A. As a proxy for systemic changes, we sampled proteins with the same technique *i.e*. microdialysis from abdominal s.c. fat. This approach also enables for distinguishing local breast tissue alterations from systemic differences. As shown in Fig. 1B, the profile of the proteins in abdominal s.c. fat from women with dense vs. nondense breast tissue was skewed in a similar fashion as the one observed in breast tissue. However, no significant changes were detected after FDR correction.

In premenopausal breast tissue compared to postmenopausal dense breasts a total of 129 proteins were significantly changed after FDR correction, *P* < 0.017, Fig. 1C and in the abdominal s.c. fat 25 proteins were significantly changed after FDR correction, *P* < 0.003, Fig. 1D.

### Breast tissue specific and systemic differences

We next identified which of the 93 proteins that were significantly changed in dense breast tissue also were significantly altered, *P* < 0.05, in abdominal s.c. fat. As shown in Supplementary Tables S1, 71 of the proteins were significantly changed in breast tissue only and 22 in both breast and abdominal s.c. fat. None of the proteins were altered in abdominal s.c. fat only.

We noted that the inflammatory proteins were skewed into a pattern of up-regulation in abdominal s.c. fat although FDR correction did not reveal any significantly changed. Because of this, we analyzed CRP in plasma to rule out any systemic inflammatory differences between these two groups of postmenopausal women. No differences were detected in levels of CRP between the groups, using a high-sensitive analysis, median (25–75 percentile), 2299 ng/ml (879–3319) vs 2698 ng/ml (1116–8671), *P* = 0.16, in the dense and nondense group respectively, Mann-Whitney test.

In premenopausal women as compared to postmenopausal women with dense breasts we identified 90 proteins that were significantly changed in breast tissue only, 39 in both breast tissue and abdominal s.c. fat, and 4 in abdominal s.c. fat only, Supplementary Table S2.

### Correlations with breast density (LTF) or local breast E2 levels

**Fig. 2:**
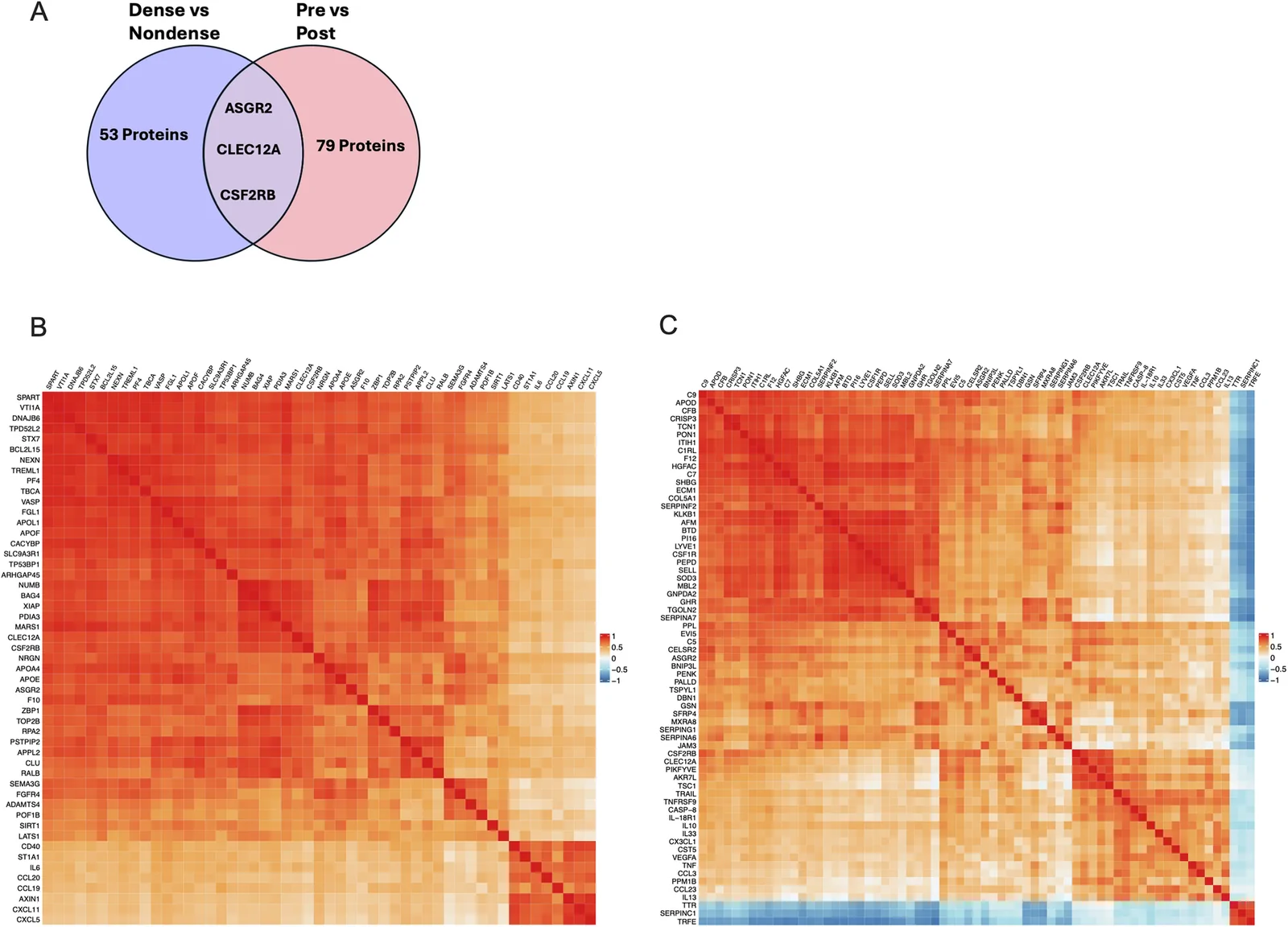
Associations of breast specific altered proteins related to breast density (LTF) or local breast estradiol (E2) levels. Microdialysis was performed as described in Fig. 1. Proteins that were significantly altered in breast tissue only and significantly associated with LTF or E2 were analyzed. **A** 56 breast specific proteins were significantly correlated with LTF and 82 with E2. Three proteins correlated both with LTF and E2. **B** Heatmap of pairwise correlated LTF-associated proteins. Significant correlations (FDR < 0.01, |ρ | ≥ 0.6) are shown for proteins with ≥ 3 significant associations. **C** Heatmap of pairwise correlated E2-associated proteins. Significant correlations (FDR < 0.01, |ρ | ≥ 0.6) are shown for proteins with ≥ 5 significant associations. Proteins are ordered by hierarchical clustering. Color scale indicates correlation strength and direction (−1 to 1).

To further explore the relationships of protein levels to LTF or local breast tissue estradiol, correlations with these two factors were analyzed. In dense versus nondense breasts, 56 of the breast specific 71 proteins exhibited a significantly, *P* < 0.05, positive correlation with LTF, whereas one protein exhibited a negative correlation. In the premenopausal versus postmenopausal women, 82 out of the 90 proteins correlated significantly with local breast E2 levels; 3 were negatively correlated and 79 positively correlated. Three proteins; ASGR2, CSF2RB, and CLEC12 A correlated with both LTF and E2 levels, Fig. 2A.

### Co-variations in LTF and E2 associated proteomes

To define the proteomic landscape associated with breast density or local breast E2, we assessed pairwise correlations between proteins using Spearman’s analysis. Significant associations (FDR < 0.01, |ρ | ≥ 0.6) revealed a structured pattern of co-variation. Hierarchical clustering identified different patterns in the two data sets. As shown in Fig. 2B, all LTF-related proteins were positively correlated whereas the E2 associated proteins distinct clusters of positively correlated proteins were revealed alongside a smaller subset of inverse correlations, Fig. 2C.

### Heterogeneity in node connectivity and centrality in network topology

**Fig. 3:**
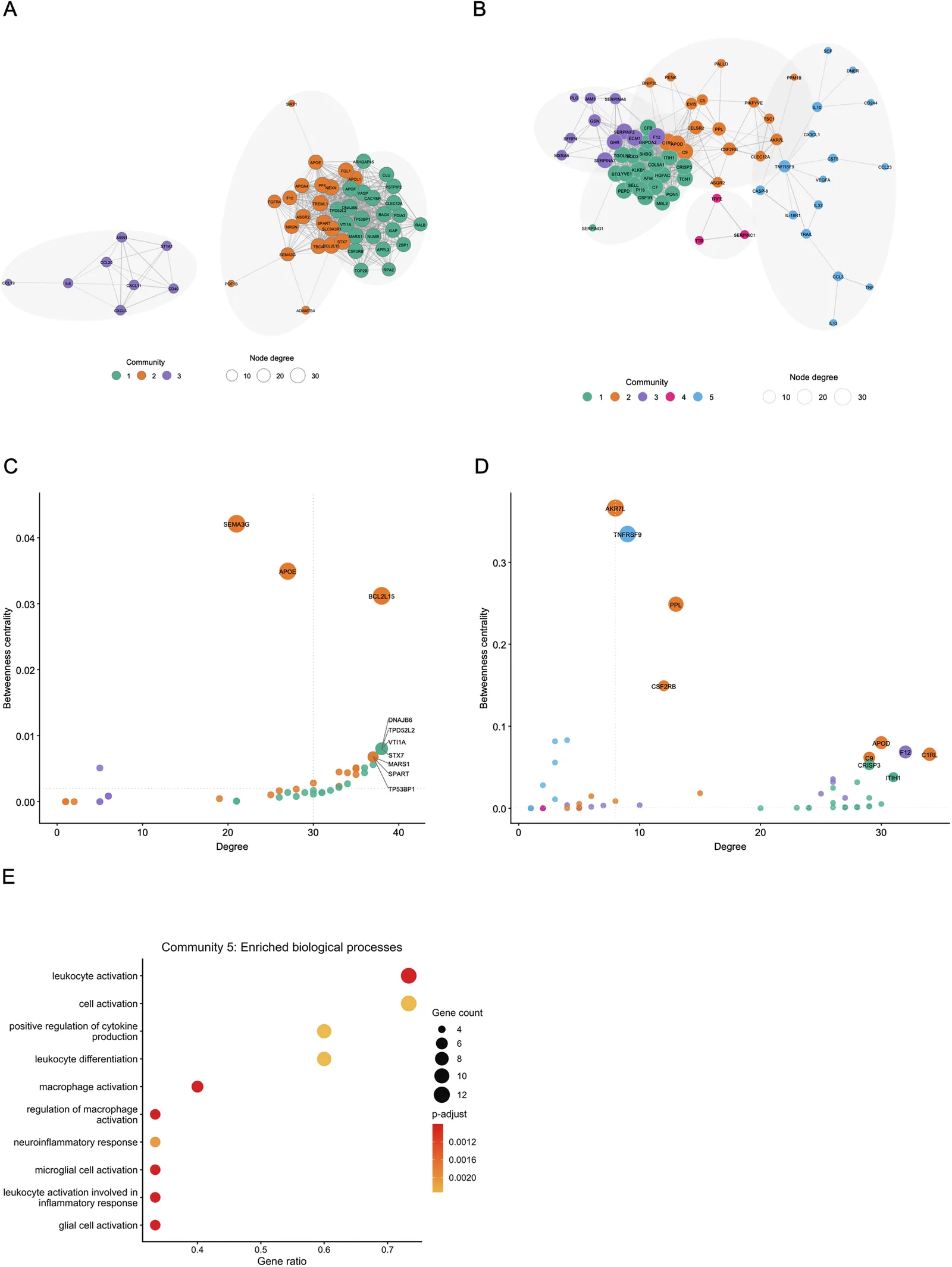
Proteomic network of proteins associated with breast density (LTF) or local breast estradiol (E2) levels. Microdialysis was performed as described in Fig. 1. Proteins that were significantly altered in breast tissue only and significantly associated with LTF or E2. Node size reflects degree and node color indicates Louvain-defined communities. **A** Correlation network of LTF-associated proteins comprising 50 proteins and 639 edges. **B** Correlation network of E2-associated proteins comprising 69 proteins and 482 edges. Spearman |ρ | > 0.7, FDR < 0.01. Nodes represent proteins and edges represent significant positive correlations. Node size reflects degree (number of connections), node color indicates Louvain-defined communities, and edge width reflects correlation strength. **C** Gene Ontology Biological Process enrichment analysis of proteins in Community 5 in the E2 group. X-axis is gene ratio, dot size represents gene count, and color indicates adjusted *P* value (Benjamini–Hochberg correction). No statistically significant pathways of the LTF-derived communities were detected after multiple-testing correction. **D** Scatter plot of node degree versus betweenness centrality in the LTF-associated network. Each point represents a protein colored by community membership. Proteins with high degree and/or betweenness centrality are labeled. Dotted lines indicate median values. **E** Scatter plot of node degree versus betweenness centrality for proteins in the E2-associated network. Each point represents a protein colored by community membership. Proteins with high connectivity and/or central positioning are labeled. Dotted lines indicate median degree and betweenness centrality.

Using more stringent criteria (|ρ | > 0.7, FDR < 0.01), we constructed correlation networks. The LTF associated proteins yielded a network of 50 proteins connected by 639 edges. Louvain clustering identified three communities, indicating a modular organization of the LTF-associated proteome, Fig. 3A. The E2 related proteins comprised 69 proteins connected by 482 edges. Louvain community detection identified five distinct clusters, indicating a modular organization of the E2-associated proteome, Fig. 3B.

Network topology analysis revealed heterogeneity in node connectivity and centrality among on both LTF and E2 associated proteins. Degree distribution varied widely, with a subset of proteins exhibiting high connectivity (degree > 30), while others showed lower connectivity. Betweenness centrality was generally low across the networks, with a limited number of proteins displaying elevated values. Of the LTF-associated proteins integration of degree and betweenness centrality identified highly connected proteins, including STX7, VTI1A, DNAJB6, and TPD52L2, and proteins with elevated betweenness centrality, including SEMA3G, APOE, and BCL2L15, Fig. 3C. Integration of these metrics in the E2-associated proteins identified highly connected proteins, including APOD, C1RL, CRISP3, and ITIH1, and proteins with elevated betweenness centrality, including AKR7L, TNFRSF9, PPL, and CSF2RB, Fig. 3D.

Functional enrichment analysis did not identify statistically significant Gene Ontology, KEGG, or Reactome pathways across any of the LTF-derived communities after multiple-testing correction. In the E2-associated proteins, communities 1, 2, and 4 did not show statistically significant enrichment for Gene Ontology (GO), KEGG, or Reactome pathways. Community 3 showed a pattern consistent with coagulation and proteolysis, although enrichment did not reach statistical significance. In contrast, community 5 showed significant enrichment for immune-related biological processes, including leukocyte activation, cytokine production, and macrophage activation, Fig. 3E.

### Correlations of breast specific proteins with LTF and E2

**Fig. 4:**
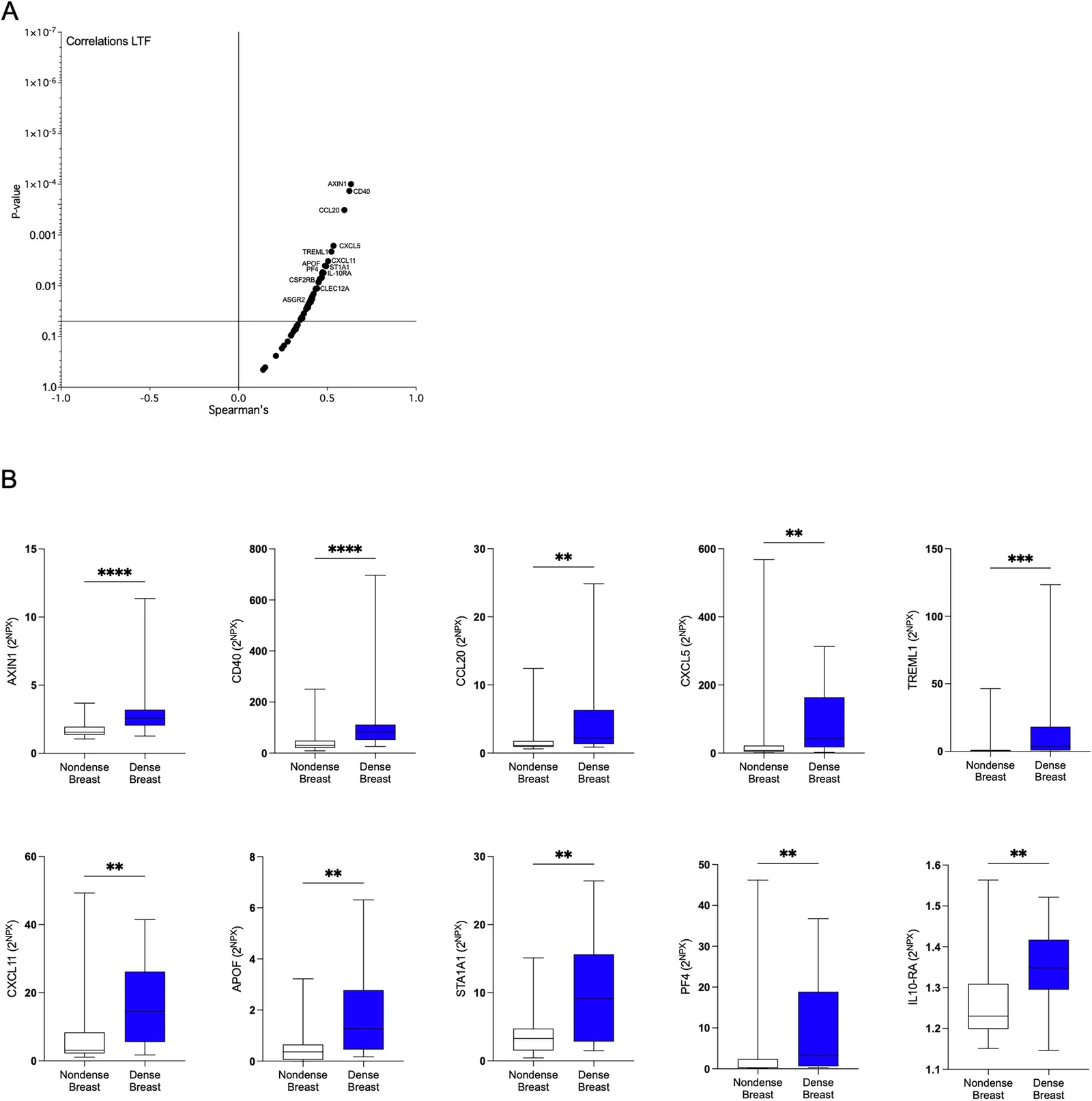
Correlations of breast tissue-specific altered proteins and breast density (LTF). Extracellular proteins were sampled using microdialysis, as described under Fig. 1. **A** Spearman correlation coefficients for the associations between LTF and proteins that were altered in breast tissue only between postmenopausal women with dense or nondense breasts. **B** Levels of the ten proteins with the strongest correlation with LTF (dense, *n* = 20, or nondense, *n* = 20). Box plots with min–max. Mann–Whitney U test, **P < 0.01, ***P < 0.001, ****P < 0.0001.

Next, we investigated the breast specific proteins associated with each risk factor, respectively. As shown in Fig. 4A, the protein with the highest correlation co-efficient with LTF was AXIN1, Spearman’s ρ = 0.633, *P* < 10^-4^. The levels in dense and nondense breast tissue of the ten proteins with the highest correlation with LTF, including AXIN1, CD40, CCL20, CXCL5, TREML1, CXCL11, APOF, ST1A1, PF4, and IL-10RA, are plotted in Fig. 4B.

**Fig. 5:**
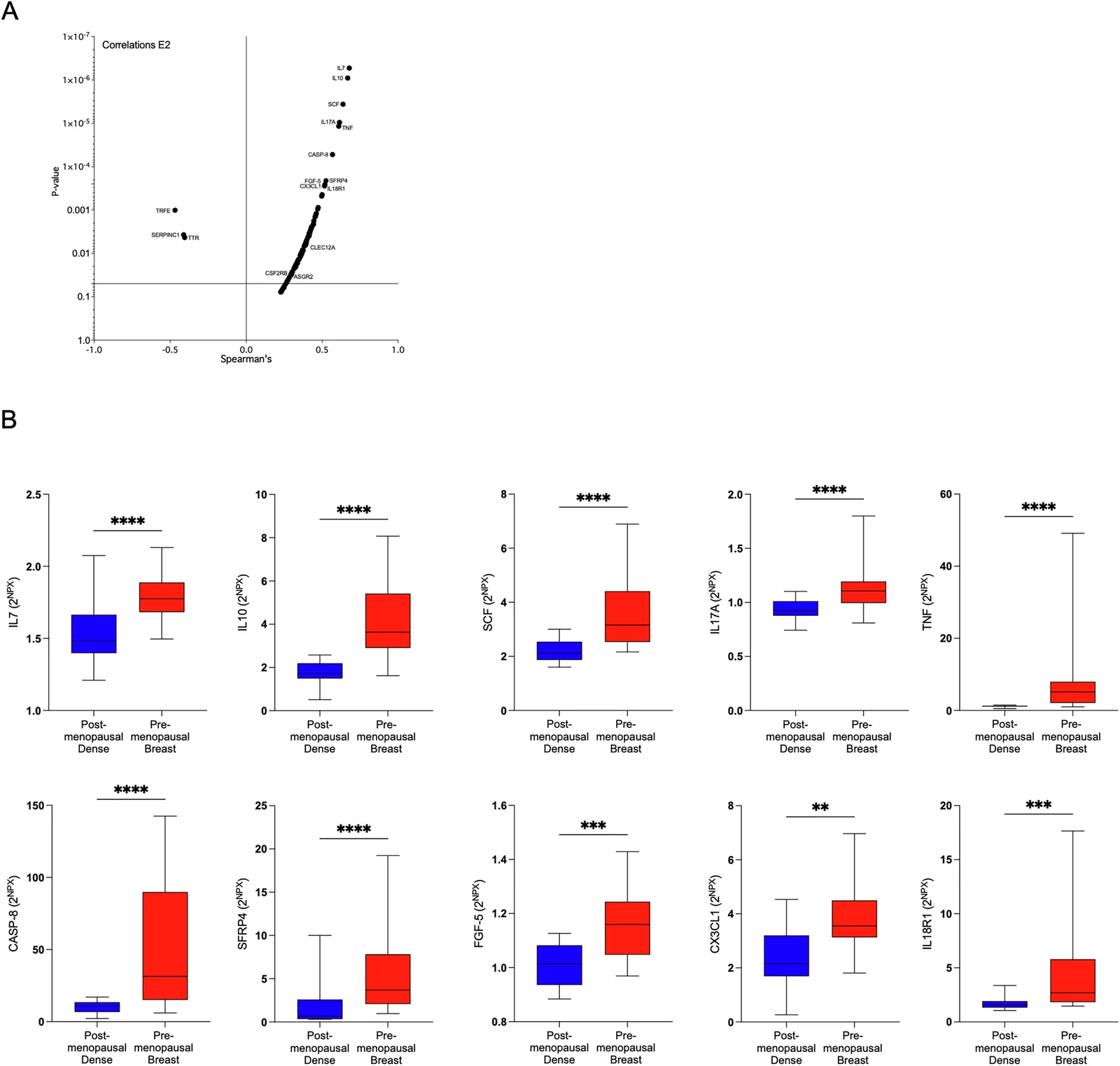
Correlations of breast tissue-specific altered proteins and local breast estradiol (E2) levels. Extracellular proteins were sampled using microdialysis, as described under Fig. 1. **A** Spearman correlation coefficients for the associations between local breast E2 levels and proteins that were altered in breast tissue only between pre- and postmenopausal women. **B** Levels of the ten proteins with the strongest correlation with breast E2 levels (*n* = 19 in the premenopausal group and *n* = 21 in the postmenopausal group). Box plots with min–max. Mann–Whitney U test, **P < 0.01, ***P < 0.001, ****P < 0.0001.

Similarly, the proteins that were associated with E2 are shown in Fig. 5A. The protein with the highest correlation coefficient with E2 was IL7, Spearman’s ρ = 0.68, *P* < 10^-6^. The levels in postmenopausal dense breasts and premenopausal breasts of the ten proteins with the highest correlation with E2, including IL7, IL10, SCF, IL17A, TNF, CASP-8, SFRP4, FGF-5, CX3CL1, and IL18R1, are depicted in Fig. 5B.

### Biological function of the up-regulated proteins

The 56 and 82 proteins that correlated with LTF and E2, respectively are grouped by biological function in Table 2.

**Table 2:** Breast specific altered proteins that correlated significantly with breast density (LTF) or local breast estradiol (E2) grouped depending on main biological function

Main biological function	LTF-associated proteins	E2-associated protein
Immune signaling	CXCL5, CXCL9, CXCL11, CCL20, IL6, PSTPIP2, ARGHGAP45, **CLEC12A**, TREML1, ZBP1, **CSF2RB**, CD40, CD7, IL10-RA, BLNK, CCL19	TNF, IL33, CCL3, CCL23, CX3CL1, CSF1R, **CLEC12A**, IL18R1, OLFM4, SCF, **CSF2RB**, TRAIL, CRISP3, IL4, IL13, IL17A, IL10, CD244, TNFRSF9, SELL
Complement		C5, C7, C9, CFB, MBL2
Angiogenesis		VEGF, FGF5, TGFBR1, ECM1, LYVE1, JAM3, APOD, SOD3, HGFAC
ECM remodeling/proteases	ADAMTS4, SEMA3G, CLU, FGL1	ADAM12, COL5A1, ITIH1, MXRA8, PALLD, SERPINF2, SERPINC1, COL5A1, DBN1, SERPINA6, SERPINA7, SERPING1, PEPD, GNPDA2, CST5
Vesicle trafficking	VTI1A, STX7, SLC9A3R1, TPD52L2	
Coagulation	F10, PF4	PLG, F12, KLKB1
Metabolism/lipid transporters	APOE, APOA4, APOF, APOL1, ST1A1, MARS1, FGFR4	GHR, BTD
Extracellular presence of intracellular proteins including tumor suppressors	AXIN1, RAP1A, RALB, LATS1, NUMB, APPL2, SIRT1, TOP2B, RPA2, TP53BP1, DNAJB6, BAG4, PDIA3, TBCA, XIAP, BCL2L15, POF1B, VASP, CACYBP	CASP8, BNIP3L, TSC1, PIKFYVE, PPM1B, TGOLN2, EVI5, BABAM1, MRPS16, PON1
Other	**ASGR2**, SPART, NRGN, NEXN	**ASGR2**, TTR, SHBG, AFM, APOD, TCN1, DNER, PENK, PPL, CELSR2, TSPYL1, SFRP4, DAND5, AKR7L, PI16, KLK7, TRFE, C1RL

The three Proteins that correlated with both parameters in **bold**.

## Discussion

Here, we demonstrate that dense and estrogen-exposed normal human breast tissues exhibit distinct inflammatory molecular signatures in situ, indicating that these two major risk factors for breast cancer are driven by fundamentally different microenvironmental processes and may require distinct preventive strategies.

The breast microenvironment is a critical determinant of cancer progression. Autopsy studies show that up to one-third of women harbor ductal carcinoma in situ (DCIS), whereas invasive breast cancer occurs in <1% of women in the same age group (40–50 years)^21^. The similar prevalence of DCIS across different breast densities further indicates that breast density does not influence tumor initiation but rather progression, underscoring the decisive role of the microenvironment^22^. Thus, there is strong evidence that the stroma determines whether DCIS progresses into invasive disease or remains indolent. Additionally, several hallmarks of cancer emphasize that interactions between epithelial cells and the surrounding stroma, rather than tumor cells alone, govern disease progression^1,23^.

Inflammation is a central regulator of tissue homeostasis with context-dependent roles in cancer. While it can promote tumor initiation and progression, it may also activate anti-tumor immunity^1^. Inflammatory mediators primarily act as soluble extracellular factors, which make their in situ assessment challenging. By applying microdialysis to live tissue, we captured extracellular proteins directly within the breast microenvironment and, by parallel sampling of subcutaneous fat, distinguished local from systemic effects.

In dense breasts, a limited but distinct subset of proteins was altered (71 out of 461), suggesting a microenvironment characterized by immune engagement and growth-regulatory signaling with minimal angiogenic activation. Notably, several upregulated proteins have established intracellular tumor-suppressive functions (e.g., AXIN1, LATS1, TP53BP1, RPA2, TOP2B, XIAP). Despite their intracellular location, all of these proteins have been shown to be detectable and quantifiable in blood from different disease cohorts, including breast cancer patients, as reported in the Human Protein Atlas (https://www.proteinatlas.org). Their extracellular detection, despite canonical intracellular roles, may indicate cellular stress or damage, resulting in the release of these proteins. Concurrently, we observed increased levels of cytokines linked to poor breast cancer outcomes (e.g., CXCL11, CCL20, IL-6, IL-10)^24–26^, indicating that this environment may combine immune activation with immunosuppressive signaling.

A prominent feature of dense breasts was the upregulation of apolipoproteins, including APOE, APOA4, APOL1, and APOF, implicating altered lipid handling. Given the central role of lipid metabolism in supporting proliferation and modulating immune responses by influencing innate immune cells such as neutrophils and macrophages, as well as T-cell function, this finding suggests a metabolically rewired microenvironment with potential immunosuppressive consequences^27–29^. APOE, which showed the strongest correlation with breast density, has been identified as an independent risk factor and is associated with decreased overall survival in breast cancer patients^30^.

In contrast, estrogen-exposed breasts exhibited a markedly broader and coordinated protein response. Eighty two out of 461 breast-specific proteins correlated with local estradiol levels. Key cytokines and immune regulators associated with tumor-promoting inflammation were consistently upregulated, indicating a shift toward a pro-tumorigenic immune milieu^1,31–35^. This inflammatory state was tightly coupled to angiogenesis. Both indirect (immune-mediated) and direct pro-angiogenic factors, including VEGF and related signaling molecules, were strongly associated with estradiol levels, indicating active vascular remodeling^36,37^.

In parallel, extensive extracellular matrix remodeling was evident through upregulation of proteins such as COL5A1, ECM1, and ADAM12, supporting a cancer permissive microenvironment. Additionally, estrogen exposure was associated with activation of the complement system, particularly components C3 and C5, which are known to drive tumor-promoting inflammation and enhance proliferation, migration, and epithelial–mesenchymal transition^38^. Together, these findings define an integrated program of inflammation, angiogenesis, and stromal remodeling in estrogen-exposed breast tissue.

Importantly, network analysis revealed that LTF-associated proteins form a highly interconnected network with low modularity, characterized by widespread positive correlations and limited functional segregation. This topology suggests a coordinated, system-level response, potentially reflecting tissue stress or remodeling rather than activation of distinct extracellular pathways. In contrast, the estrogen-associated proteome displayed a more modular and functionally segregated network architecture, comprising multiple distinct communities. While only one community showed statistically significant enrichment for immune-related processes, the overall modular organization suggests parallel activation of multiple biological programs, including immune signaling and tissue remodeling, indicative of a more structured and multi-layered microenvironment associated with estrogen exposure.

Taken together, our data reveal two fundamentally different microenvironmental states linked to breast cancer risk. Dense breast tissue is characterized by features consistent with cellular stress, immune modulation, and altered lipid metabolism, with limited activation of angiogenesis and matrix remodeling. In contrast, estrogen-exposed tissue displays a coordinated pro-inflammatory and pro-angiogenic program, coupled to stromal and complement activation, hallmarks of a microenvironment that actively supports tumor progression.

These distinctions may have important implications. Our findings provide mechanistic insight and suggest that prevention should be stratified according to the underlying microenvironmental phenotype. Targeting metabolic and stress-related pathways may be more relevant in dense breasts, whereas anti-inflammatory, anti-angiogenic, or stromal-directed approaches may be required in estrogen-driven risk. By defining breast tissue–specific protein signatures in situ, this study offers insight to guide the development of prevention strategies and highlights candidate pathways for targeted intervention.

## Methods

### Subjects

Previously collected and biobanked samples from different cohorts were used in the exploratory clinical study. The Regional Ethical Review Board of Linköping approved the collections (approval numbers 2014/185-32 and M61-05/32-06), which were carried out in accordance with the Declaration of Helsinki. All subjects gave informed consent. A total of 61 women were included.

Healthy postmenopausal women from the mammography screening program at Linköping University Hospital that were categorized according to the Breast Imaging Reporting and Data System (BI-RADS) as either entirely fatty nondense breasts (BI-RADS A) or extremely dense breasts (BI-RADS D) were invited to the study^39^. Forty-two healthy postmenopausal women (ages 55–74 years) were consecutively recruited for the study. The women were subjected to magnetic resonance imaging (MRI)^40,41^. As a continuous measure of breast density, lean tissue fraction (LTF), was calculated on MRIs in a volume selection of 20 × 20 × 20 mm in the upper lateral quadrant of the left breast, as previously described^40,42^. In brief, 1.5 T Achieva MR scanner (Philips Healthcare, Best, Netherlands) using a dual breast seven-element breast coil was used. Water- and fat separated MR images were computed, as previously described^43^, in summary: axial 3D 6-echo turbo field echo MRI images, anterior-posterior frequency encoding, first TE at 2.3 ms and ΔTE of 2.3 ms, TR 15.4 ms, flip angle 10°, 300 × 300 × 150 mm^3^ field of view, 200 × 200 scan matrix and 3 mm slice thickness. LTF was computed as the ratio of lean tissue volume to total volume.

A second review of the mammograms revealed that two women had been miscategorized on the BI-RADS scale. These two women were not included in the analyses of dense vs. nondense breasts but were included in the correlation analyses.

19 nulliparous women (ages 20–32 years) with a history of regular menstrual cycles (cycle length, 27–34 days) were included in the premenopausal group. All of these women were investigated with microdialysis in the luteal phase of the menstrual cycle.

None of the healthy volunteer women had a history of breast cancer or were currently using (or had used within the past 3 months) hormone replacement therapy, sex steroid-containing contraceptives, anti-estrogen therapies, including selective estrogen receptor modulators, or degraders. None of the premenopausal women had other ongoing medications. In the postmenopausal group antihypertensives, statins, and SSRI were allowed.

### Microdialysis procedure

Prior to insertion of the microdialysis catheters 0.5 mL lidocaine (10 mg*/*mL) was administered intracutaneously. Microdialysis catheters (M Dialysis AB, Stockholm, Sweden), which consisted of a 20 mm long tubular dialysis membrane (diameter 0.52 mm, 100,000 atomic mass cut-off) glued to the end of a double-lumen tube were inserted via a splitable introducer (M Dialysis AB), connected to a microinfusion pump (M Dialysis AB) and perfused with 154 mmol*/*L NaCl and 60 g/L hydroxyethyl starch (Voluven®; Fresenius Kabi, Uppsala, Sweden), at 0.5 µL/min. One catheter was placed in the upper lateral quadrant of the left breast and directed towards the nipple and one catheter was inserted subcutaneously s.c in abdominal fat tissue, as previously described^13,44–51^. The breast density category in the upper lateral quadrant of the left breast, BI-RADS A or BI-RADS D, was confirmed by MRI prior insertion of the microdialysis catheters.

After a 60 min equilibration period, the outgoing perfusate was stored at -80°C for subsequent analysis.

### Protein quantifications

The microdialysates were analyzed with multiplex proximity extension assay (PEA, Olink Bioscience, Uppsala Sweden) as previously described^52–54^. A total of 461 unique inflammatory proteins were quantified using specific antibodies, with the Target 96 Inflammation and Explore Inflammation II panels.

In brief, 1 μL sample was incubated with proximity antibody pairs tagged with DNA reporter molecules. The DNA tails formed an amplicon by proximity extension, which was quantified by high-throughput real-time PCR (BioMark™ HD System; Fluidigm Corporation, South San Francisco, CA, USA). The generated fluorescent signal correlates with protein abundance by quantitation cycles (Cq) produced by the BioMark Real-Time PCR Software. To minimize variation within and between runs, the data were normalized using both an internal control (extension control) and an interplate control and transformed using a predetermined correction factor. The pre-processed data were provided in the arbitrary unit normalized protein expression (NPX) on a log_2_ scale, which were then linearized by using the formula 2^NPX^. A high NPX value corresponds to high protein concentrations. Values represented a relative quantification meaning that no comparison of absolute levels between different proteins could be made.

### Estradiol and human c-reactive protein (CRP) analyses

Estradiol in microdialysates was analyzed using a high sensitivity immunoassay kit (DRG International, Springfield Township, NJ, USA) and CRP in plasma with Quantikine Elisa kit (Bio-Techne, MN, USA, Cat# DCRP00B) assayed using VersaMax™ Microplate Reader (Molecular Devices, Sunnyvale, CA, USA).

### Statistical analyses

Statistical analyses were performed using two-sided nonparametric unpaired Mann-Whitney U tests. Spearman’s correlation test was used for calculations of correlations. A *P* < 0.05 was considered statistically significant. False discovery rate (FDR) was performed using two-stage set-up method of Benjamini, Krieger and Yekutieli which was set to 5%. Statistics were performed with Prism 10.6 (GraphPad, San Diego, CA, USA).

For pairwise protein–protein associations, Spearman rank correlation coefficients (ρ) were used. Significant correlations were defined as FDR < 0.01 and |ρ | ≥ 0.60 using the Benjamini–Hochberg. Connectivity was defined as the number of significant associations per protein, and proteins were retained using dataset-specific thresholds (E2: ≥5; LTF: ≥3). Correlation matrices were visualized using hierarchical clustering and a diverging color scale (−1 to 1).

Correlation networks were constructed from significant associations (|ρ | > 0.7, FDR < 0.01) to focus on strong interactions. Node-level metrics, including degree and normalized betweenness centrality, were calculated to characterize network topology. Community structure was identified using the Louvain algorithm. Network visualization was performed using a stress-based layout, with node size scaled by degree and node color indicating community membership.

To identify hub proteins, degree and betweenness centrality were standardized (z-scores) and summed to generate a composite hub score. Proteins were ranked based on this score, and the top 10 proteins were selected for annotation. Functional enrichment analysis was performed using Gene Ontology Biological Process annotations with a custom background of all quantified proteins (*n* = 461). Enrichment significance was defined as adjusted *P* < 0.05 (Benjamini–Hochberg correction). Community-level enrichment analyses were conducted using the same approach. KEGG and Reactome analyses were performed but did not yield significant results.

All analyses were performed in RStudio (RRID:SCR_000432) using standard packages including *ComplexHeatmap*, *igraph*, *tidygraph*, *ggraph*, *ggplot2*, *clusterProfiler*, and *ReactomePA*.

## Supplementary information

Suppementary Table S1 and S2

## Data Availability

The datasets analyzed during the current study are not publicly available due to participant confidentiality but are available from the corresponding author on reasonable request.

## References

[1] Hanahan, D. & Weinberg, R. A. Hallmarks of cancer: the next generation. *Cell* **144**, 646–674 (2011). 21376230 10.1016/j.cell.2011.02.013

[2] Lim S. Y., Yuzhalin A. E., Gordon-Weeks A. N., Muschel R. J. Tumor-infiltrating monocytes/macrophages promote tumor invasion and migration by upregulating S100A8 and S100A9 expression in cancer cells. Oncogene. 2016. 10.1038/onc.2016.107 PMC4961254 27086923

[3] Wculek, S. K. & Malanchi, I. Neutrophils support lung colonization of metastasis-initiating breast cancer cells. *Nature* **528**, 413–417 (2015). 26649828 10.1038/nature16140 PMC4700594

[4] Lindahl, G., Saarinen, N., Abrahamsson, A. & Dabrosin, C. Tamoxifen, flaxseed, and the lignan enterolactone increase stroma- and cancer cell-derived IL-1Ra and decrease tumor angiogenesis in estrogen-dependent breast cancer. *Cancer Res.* **71**, 51–60 (2011). 21097717 10.1158/0008-5472.CAN-10-2289

[5] Balkwill, F. Tumour necrosis factor and cancer. *Nat. Rev. Cancer* **9**, 361–371 (2009). 19343034 10.1038/nrc2628

[6] Yu, P. F. et al. TNFalpha-activated mesenchymal stromal cells promote breast cancer metastasis by recruiting CXCR2(+) neutrophils. *Oncogene* **36**, 482–490 (2017). 27375023 10.1038/onc.2016.217 PMC5290040

[7] Bray, F. et al. Global cancer statistics 2018: GLOBOCAN estimates of incidence and mortality worldwide for 36 cancers in 185 countries. *CA Cancer J. Clin.* **68**, 394–424 (2018). 30207593 10.3322/caac.21492

[8] Kaaks, R. et al. Postmenopausal serum androgens, oestrogens and breast cancer risk: the European prospective investigation into cancer and nutrition. *Endocr. Relat. Cancer* **12**, 1071–1082 (2005). 16322344 10.1677/erc.1.01038

[9] Boyd, N. F. et al. Breast tissue composition and susceptibility to breast cancer. *J. Natl. Cancer Inst.* **102**, 1224–1237 (2010). 20616353 10.1093/jnci/djq239 PMC2923218

[10] Anderson, T. J., Ferguson, D. J. & Raab, G. M. Cell turnover in the “resting” human breast: influence of parity, contraceptive pill, age and laterality. *Br. J. Cancer* **46**, 376–382 (1982). 7126427 10.1038/bjc.1982.213 PMC2011125

[11] Wilson, C. L., Sims, A. H., Howell, A., Miller, C. J. & Clarke, R. B. Effects of oestrogen on gene expression in epithelium and stroma of normal human breast tissue. *Endocr. Relat. Cancer* **13**, 617–628 (2006). 16728587 10.1677/erc.1.01165

[12] Shekhar, M. P., Werdell, J., Santner, S. J., Pauley, R. J. & Tait, L. Breast stroma plays a dominant regulatory role in breast epithelial growth and differentiation: implications for tumor development and progression. *Cancer Res.* **61**, 1320–1326 (2001). 11245428

[13] Bendrik, C. & Dabrosin, C. Estradiol increases IL-8 secretion of normal human breast tissue and breast cancer in vivo. *J. Immunol.* **182**, 371–378 (2009). 19109168 10.4049/jimmunol.182.1.371

[14] Garvin, S., Nilsson, U. W., Huss, F. R., Kratz, G. & Dabrosin, C. Estradiol increases VEGF in human breast studied by whole-tissue culture. *Cell Tissue Res.* **325**, 245–251 (2006). 16568303 10.1007/s00441-006-0159-7

[15] Svensson, S. et al. CCL2 and CCL5 Are Novel Therapeutic Targets for Estrogen-Dependent Breast Cancer. *Clin. Cancer Res* **21**, 3794–3805 (2015). 25901081 10.1158/1078-0432.CCR-15-0204

[16] Vazquez Rodriguez, G., Abrahamsson, A., Jensen, L. D. & Dabrosin, C. Estradiol Promotes Breast Cancer Cell Migration via Recruitment and Activation of Neutrophils. *Cancer Immunol. Res.* **5**, 234–247 (2017). 28159748 10.1158/2326-6066.CIR-16-0150

[17] Chlebowski, R. T. & Geller, M. L. Adherence to endocrine therapy for breast cancer. *Oncology* **71**, 1–9 (2006). 17344666 10.1159/000100444

[18] Hawes, D. et al. Dense breast stromal tissue shows greatly increased concentration of breast epithelium but no increase in its proliferative activity. *Breast Cancer Res.* **8**, R24 (2006). 16646977 10.1186/bcr1408 PMC1557710

[19] Alowami, S., Troup, S., Al-Haddad, S., Kirkpatrick, I. & Watson, P. H. Mammographic density is related to stroma and stromal proteoglycan expression. *Breast Cancer Res.* **5**, R129–35 (2003). 12927043 10.1186/bcr622 PMC314426

[20] Khan, Q. J. et al. Mammographic density does not correlate with Ki-67 expression or cytomorphology in benign breast cells obtained by random periareolar fine needle aspiration from women at high risk for breast cancer. *Breast Cancer Res.* **9**, R35 (2007). 17537236 10.1186/bcr1683 PMC1929099

[21] Welch, H. G. & Black, W. C. Using autopsy series to estimate the disease “reservoir” for ductal carcinoma in situ of the breast: how much more breast cancer can we find? *Ann. Intern Med* **127**, 1023–1028 (1997). 9412284 10.7326/0003-4819-127-11-199712010-00014

[22] Bertrand, K. A. et al. Mammographic density and risk of breast cancer by age and tumor characteristics. *Breast Cancer Res* **15**, R104 (2013). 24188089 10.1186/bcr3570 PMC3978749

[23] Kim, J. & DeBerardinis, R. J. Mechanisms and Implications of Metabolic Heterogeneity in Cancer. *Cell Metab.* **30**, 434–446 (2019). 31484055 10.1016/j.cmet.2019.08.013 PMC6730674

[24] Kong T., Ma C. X. Unraveling TIME: CD8+ T cell- and CXCL11-driven endocrine resistance in breast cancer. J Clin Invest. 2026;136. 10.1172/JCI200923 PMC12867142 41623177

[25] Kwantwi, L. B., Boafo, J. D., Egleh, B. E. & Li, M. CCL20 in the tumor microenvironment: implications for cancer progression and therapeutic approaches. *Clin. Transl. Oncol.* **27**, 3285–3292 (2025). 39985603 10.1007/s12094-025-03874-5 PMC12259718

[26] Castro-Espin, C. et al. Prognostic role of pre-diagnostic circulating inflammatory biomarkers in breast cancer survival: evidence from the EPIC cohort study. *Br. J. Cancer* **131**, 1496–1505 (2024). 39342063 10.1038/s41416-024-02858-6 PMC11519559

[27] Darwish N. M., et al. The Role of Apolipoproteins in the Commonest Cancers: A Review. Cancers (Basel). 2023;15. 10.3390/cancers15235565 PMC10705282 38067270

[28] Lin, J. et al. Oncogene APOL1 promotes proliferation and inhibits apoptosis via activating NOTCH1 signaling pathway in pancreatic cancer. *Cell Death Dis.* **12**, 760 (2021). 34341330 10.1038/s41419-021-03985-1 PMC8329288

[29] Huang, B., Song, B. L. & Xu, C. Cholesterol metabolism in cancer: mechanisms and therapeutic opportunities. *Nat. Metab.* **2**, 132–141 (2020). 32694690 10.1038/s42255-020-0174-0

[30] Xu, X. et al. Serum levels of apolipoprotein E correlates with disease progression and poor prognosis in breast cancer. *Tumour Biol.* **37**, 15959–15966 (2016). 27709551 10.1007/s13277-016-5453-8

[31] Coussens, L. M., Zitvogel, L. & Palucka, A. K. Neutralizing tumor-promoting chronic inflammation: a magic bullet? *Science* **339**, 286–291 (2013). 23329041 10.1126/science.1232227 PMC3591506

[32] Mantovani, A., Allavena, P., Sica, A. & Balkwill, F. Cancer-related inflammation. *Nature* **454**, 436–444 (2008). 18650914 10.1038/nature07205

[33] Giotopoulou, N., Shi, W., Parniewska, M. M., Sun, W. & Fuxe, J. TGFss1 Stimulates Lymphatic Endothelial Cells to Produce IL7 and IL15, Which Act as Chemotactic Factors for Breast Cancer Cells with Mesenchymal Properties. *J. Mammary Gland Biol. Neoplasia* **28**, 25 (2023). 38055067 10.1007/s10911-023-09552-y PMC10700205

[34] Karihtala, P. et al. Serum protein profiling reveals an inflammation signature as a predictor of early breast cancer survival. *Breast Cancer Res.* **26**, 61 (2024). 38594742 10.1186/s13058-024-01812-x PMC11005292

[35] Song, J. & Yang, H. Identifying new biomarkers and potential therapeutic targets for breast cancer through the integration of human plasma proteomics: a Mendelian randomization study and colocalization analysis. *Front Endocrinol. (Lausanne)* **15**, 1449668 (2024). 39351539 10.3389/fendo.2024.1449668 PMC11439655

[36] Fox, S. B., Gasparini, G. & Harris, A. L. Angiogenesis: pathological, prognostic, and growth-factor pathways and their link to trial design and anticancer drugs. *Lancet Oncol.* **2**, 278–289 (2001). 11905783 10.1016/S1470-2045(00)00323-5

[37] Cao, Y., Langer, R. & Ferrara, N. Targeting angiogenesis in oncology, ophthalmology and beyond. *Nat. Rev. Drug Discov.* **22**, 476–495 (2023). 37041221 10.1038/s41573-023-00671-z

[38] Afshar-Kharghan, V. The role of the complement system in cancer. *J. Clin. Invest* **127**, 780–789 (2017). 28248200 10.1172/JCI90962 PMC5330758

[39] Sickles E., et al. ACR BI-RADS® Mammography. In: ACR BI-RADS® Atlas, Breast Imaging Reporting and Data System. Reston, VA: American College of Radiology; 2013.

[40] Abrahamsson, A. et al. Dense breast tissue in postmenopausal women is associated with a pro-inflammatory microenvironment in vivo. *Oncoimmunology* **5**, e1229723 (2016). 27853653 10.1080/2162402X.2016.1229723 PMC5087296

[41] Lundberg, P. et al. Breast density is strongly associated with multiparametric magnetic resonance imaging biomarkers and pro-tumorigenic proteins in situ. *Br. J. Cancer* **127**, 2025–2033 (2022). 36138072 10.1038/s41416-022-01976-3 PMC9681775

[42] Ekstrand, J. et al. Breast Density and Estradiol Are Major Determinants for Soluble TNF-TNF-R Proteins in vivo in Human Breast Tissue. *Front Immunol.* **13**, 850240 (2022). 35432372 10.3389/fimmu.2022.850240 PMC9005790

[43] Yu, H. et al. Multiecho water-fat separation and simultaneous R2* estimation with multifrequency fat spectrum modeling. *Magn. Reson Med* **60**, 1122–1134 (2008). 18956464 10.1002/mrm.21737 PMC3070175

[44] Aberg, U. W. et al. Tamoxifen and flaxseed alter angiogenesis regulators in normal human breast tissue in vivo. *PLoS One* **6**, e25720 (2011). 21984941 10.1371/journal.pone.0025720 PMC3184168

[45] Dabrosin, C. Increase of free insulin-like growth factor-1 in normal human breast in vivo late in the menstrual cycle. *Breast Cancer Res. Treat.* **80**, 193–198 (2003). 12908822 10.1023/A:1024575103524

[46] Dabrosin, C. Increased extracellular local levels of estradiol in normal breast in vivo during the luteal phase of the menstrual cycle. *J. Endocrinol.* **187**, 103–108 (2005). 16214945 10.1677/joe.1.06163

[47] Dabrosin, C. Sex steroid regulation of angiogenesis in breast tissue. *Angiogenesis* **8**, 127–136 (2005). 16211362 10.1007/s10456-005-9002-0

[48] Garvin, S. & Dabrosin, C. In vivo measurement of tumor estradiol and vascular endothelial growth factor in breast cancer patients. *BMC Cancer* **8**, 73 (2008). 18366667 10.1186/1471-2407-8-73 PMC2279135

[49] Nilsson, U. W., Abrahamsson, A. & Dabrosin, C. Angiogenin regulation by estradiol in breast tissue: tamoxifen inhibits angiogenin nuclear translocation and antiangiogenin therapy reduces breast cancer growth in vivo. *Clin. Cancer Res.* **16**, 3659–3669 (2010). 20501617 10.1158/1078-0432.CCR-10-0501

[50] Abrahamsson, A. & Dabrosin, C. Tissue specific expression of extracellular microRNA in human breast cancers and normal human breast tissue in vivo. *Oncotarget* **6**, 22959–22969 (2015). 26008976 10.18632/oncotarget.4038 PMC4673212

[51] Dabrosin, C., Hallstrom, A., Ungerstedt, U. & Hammar, M. Microdialysis of human breast tissue during the menstrual cycle. *Clin. Sci. (Lond.)* **92**, 493–496 (1997). 9176023 10.1042/cs0920493

[52] Abrahamsson, A., Rodriguez, G. V. & Dabrosin, C. Fulvestrant-Mediated Attenuation of the Innate Immune Response Decreases ER(+) Breast Cancer Growth In Vivo More Effectively than Tamoxifen. *Cancer Res.* **80**, 4487–4499 (2020). 32855207 10.1158/0008-5472.CAN-20-1705

[53] Abrahamsson, A., Rzepecka, A. & Dabrosin, C. Equal Pro-inflammatory Profiles of CCLs, CXCLs, and Matrix Metalloproteinases in the Extracellular Microenvironment In Vivo in Human Dense Breast Tissue and Breast Cancer. *Front Immunol.* **8**, 1994 (2017). 29387062 10.3389/fimmu.2017.01994 PMC5776019

[54] Mijic, S. & Dabrosin, C. Platelet Activation In Situ in Breasts at High Risk of Cancer: Relationship with Mammographic Density and Estradiol. *J. Clin. Endocrinol. Metab.* **106**, 485–500 (2021). 33180937 10.1210/clinem/dgaa820

