# Supplementary material for: Proteomic signatures identify significantly different inflammatory breast tissue microenvironments depending on mammographic density or estrogen exposure
Source: NPJ Breast Cancer. 2026 Sep 4;12:118. doi: 10.1038/s41523-026-01046-4 (PMC13545232; doi:10.1038/s41523-026-01046-4)
Supplement: Supplementary file 1 [file 41523_2026_1046_MOESM1_ESM.pdf]

Supplementary Table S1. Extracellular proteins in breast and subcutaneous (s.c.) fat. Extracellular proteins that were signigficantly altered in breast tissue only, in both breast and s.c. fat, or in s.c. fat only in postmenopausal women with dense vs nondense breasts

**Altered proteins in breast tissue only**			**Altered proteins in both breast and s.c fat**					**Altered proteins in s.c fat only**
**Protein**	**Fold change**	**p-value**	**Protein**	**Fold change breast**	**p-value breast**	**Fold change s.c. fat**	**p-value**	**Protein**
**ADAMTS4**	2.7	0.0003	**CASP-8**	1.8	0.01	1.8	0.01	**None**
**APOA4**	2.4	0.003	**CCL11**	2.5	0.001	2.1	0.004	
**APOE**	3.4	0.006	**CCL23**	2.9	0.00006	2.8	0.0008	
**APOF**	3.5	0.001	**CCL25**	2.6	0.0005	2.3	0.001	
**APOL1**	6.6	0.008	**CHAD**	1.7	0.00008	1.6	0.02	
**APPL2**	2.7	0.005	**CST1**	5.2	0.006009	3.5	0.049	
**ARHGAP45**	1.9	0.008	**CST5**	1.9	0.003	1.7	0.02	
**ASGR2**	3.5	0.0006	**CXCL1**	3.8	0.0007	3.1	0.002	
**AXIN1**	1.6	0.00004	**CXCL6**	6.4	0.0008	2.6	0.03	
**BAG4**	5.5	0.001	**FGF-19**	3.1	0.00007	3.0	0.001	
**BCL2L15**	8.6	0.01	**FGF-21**	1.3	0.001	1.2	0.04	
**BLNK**	1.9	0.007	**FGF-23**	1.2	0.0003	1.1	0.04	
**CACYBP**	3.9	0.003	**IL33**	1.7	0.0007	2.0	0.02	
**CCL19**	2.7	0.008	**IL8**	3.5	0.002	3.1	0.008	
**CCL20**	2.1	0.004	**MCP-2**	2.3	0.01	3.7	0.004	
**CD300A**	3.2	0.005	**MCP-4**	1.9	0.002	1.8	0.001	
**CD40**	2.7	0.00008	**MMP-1**	6.3	0.003	2.5	0.01	
**CD7**	2.5	0.006	**MMP-10**	1.2	0.005	1.3	0.001	
**CEBPA**	2.4	0.002	**SAA4**	2.3	0.004	1.8	0.04	
**CELSR2**	4.6	0.005	**TNFRSF9**	1.5	0.004	1.4	0.004	
**CFHR4**	7.6	0.01	**TRAIL**	1.3	0.001	1.8	0.01	
**CLEC12A**	2.4	0.0005	**VEGFA**	2.8	0.0005	2.1	0.002	
**CLU**	3.9	0.003						
**CPA4**	3.9	0.01						
**CSF2RB**	3.1	0.001						
**CXCL11**	4.6	0.003						
**CXCL5**	5.8	0.002						
**CXCL9**	1.8	0.01						
**DGKA**	1.6	0.005						
**DNAJB6**	5.3	0.004						
**F10**	1.6	0.004						
**FGFR4**	2.6	0.0009						
**FGL1**	3.2	0.007						
**IL-10RA**	1.1	0.004						
**IL6**	2.1	0.003						

**LATS1**	2.2	0.002
**MARS1**	4.1	0.005
**MTDH**	5.5	0.003
**NAGA**	2.9	0.01
**NEXN**	6.1	0.002
**NRGN**	3.6	0.0001
**NUMB**	3.9	0.001
**PDIA3**	10.4	0.002
**PENK**	2.8	0.005
**PF4**	11.6	0.001
**PNLIP**	3.3	0.01
**POF1B**	3.3	0.0003
**PSTPIP2**	2.7	0.008
**PTGES2**	2.6	0.002
**RALB**	3.8	0.002
**RAP1A**	1.8	0.005
**RPA2**	1.8	0.001
**SEMA3G**	3.7	0.005
**SIRT1**	1.2	0.006
**SLC9A3R1**	3.9	0.0007
**SPART**	5.8	0.003
**ST1A1**	2.8	0.0061
**STX7**	5.2	0.003
**SUMF1**	4.2	0.006
**SUSD5**	5.2	0.005
**TBCA**	3.7	0.006
**TOP2B**	4.7	0.001
**TP53BP1**	1.4	0.006
**TPD52L2**	5.1	0.007
**TREML1**	16.0	0.0007
**VAMP8**	2.7	0.002
**VASP**	8.7	0.003
**VTI1A**	2.8	0.001
**XIAP**	3.2	0.001
**YY1**	1.9	0.002
**ZBP1**	2.5	0.01

Supplementary Table S2. Extracellular proteins in breast and subcutaneous (s.c) fat. Extracellular proteins that were signigficantly altered in breast tissue only, in both breast and s.c. fat. or in s.c. fat only in premenopausal women vs postmenopausal women with dense breasts

**Altered proteins in breast tissue only**			**Altered proteins in both breast and s.c. fat**					**Altered proteins in s.c. fat only**		
**Protein**	**Fold change**	**p-value**	**Protein**	**Fold change breast**	**p-value breast**	**Fold change s.c. fat**	**p-value s.c. fat**	**Protein**	**Fold change s.c. fat**	**p-value**
**ADAM12**	2.4	0.0001	**ARTN**	0.8	0.002	0.6	0.000003	**Flt3L**	0.2	0.00017
**ADGRD1**	2.9	0.017	**Beta-NGF**	1.2	0.00008	1.1	0.0004	**IL-10RB**	0.7	0.000017
**AFM**	2.5	0.006	**CCL19**	9.2	0.00000006	9.4	0.0000001	**LIF-R**	0.8	0.00003
**AKR7L**	3.9	0.0017	**CCL20**	166.0	0.0000000003	105.4	0.000000001	**SLAMF1**	0.7	0.0000006
**APOD**	3.2	0.001	**CCL28**	1.5	0.002	0.8	0.01			
**ASGR2**	2.1	0.012	**CCL4**	12.5	0.000004	1.8	0.02			
**BABAM1**	1.8	0.006	**CDCP1**	1.2	0.00007	1.1	0.02			
**BLNK**	1.4	0.0098	**CXCL1**	3.5	0.001	4.1	0.0002			
**BMPER**	1.9	0.0098	**CXCL10**	43.0	0.0000001	8.8	0.00000002			
**BNIP3L**	1.8	0.003	**CXCL5**	3.8	0.005	7.6	0.0005			
**BTD**	2.2	0.002	**CXCL6**	3,00	0.0006	7.1	0.0005			
**C1RL**	2.8	0.002	**DBH**	10.8	0.004	3.2	0.02			
**C5**	2.0	0.002	**EN-RAGE**	7.2	0.007	12.0	0.000004			
**C7**	2.9	0.003	**GDNF**	1.3	0.0000001	1.2	0.006			
**C9**	2.8	0.0007	**HGF**	2.8	0.00001	1.8	0.006			
**CASP-8**	3.8	0.000004	**IL-1 alpha**	22.2	0.00000002	7.2	0.0000000004			
**CCL23**	1.6	0.003	**IL-10RA**	1.2	0.000000001	1.1	0.0009			
**CCL3**	6.7	0.0001	**IL-15RA**	0.9	0.001	0.8	0.000001			
**CD244**	1.3	0.0003	**IL36G**	4.3	0.007	2.0	0.04			
**CELSR2**	2.6	0.003	**IL6**	6.1	0.000000004	11.5	0.0000000004			
**CFB**	1.5	0.0004	**IL8**	4.2	0.0001	3.2	0.0002			
**CLEC12A**	4.0	0.0007	**LIF**	18.4	0.00000000003	25.2	0.0000000001			
**COL5A1**	3.1	0.0007	**MCP-1**	1.2	0.006	1.3	0.0003			
**CPB2**	1.3	0.016	**MCP-2**	3.4	0.01	2.9	0.001			
**CRISP3**	3.0	0.001	**MCP-3**	7.4	0.000000005	6.8	0.00000002			
**CSF1R**	7.1	0.008	**MMP-10**	4.9	0.00000000003	2.9	0.00004			
**CSF2RB**	3.6	0.01	**NT-3**	0.8	0.004	0.8	0.000009			
**CST5**	1.6	0.0008	**OPG**	0.7	0.008	0.5	0.000007			
**CX3CL1**	1.6	0.001	**ORM1**	0.8	0.005	0.8	0.002			
**DAND5**	1.4	0.005	**OSM**	9.5	0.0000002	6.9	0.00007			
**DBN1**	1.6	0.007	**PD-L1**	3.1	0.00000000003	2.0	0.0000002			
**DNER**	1.2	0.002	**PTPN9**	1.8	0.01	1.3	0.03			
**ECM1**	4.8	0.0004	**RLN2**	22.8	0.000002	4.1	0.004			
**EPHA4**	2.4	0.005	**ST1A1**	2.7	0.01	2.8	0.01			

**EVI5**	1.7	0.016
**F12**	2.0	0.0002
**FGF-5**	1.1	0.0004
**GHR**	1.7	0.008
**GNPDA2**	2.0	0.01
**GSN**	2.4	0.002
**HGFAC**	5.1	0.004
**IL-17A**	1.2	0.0002
**IL-18R1**	2.0	0.00002
**IL-20RA**	1.1	0.003
**IL10**	2.1	0.00000001
**IL13**	2.3	0.0003
**IL33**	1.5	0.002
**IL4**	1.2	0.006
**IL7**	1.2	0.00003
**ITIH1**	4.5	0.001
**JAM3**	2.4	0.002
**KLK7**	1.7	0.02
**KLKB1**	2.9	0.01
**LYVE1**	6.7	0.001
**MBL2**	4.5	0.005
**MRPS16**	1.3	0.01
**MXRA8**	2.3	0.002
**OLFM4**	2.3	0.002
**PALLD**	2.5	0.01
**PENK**	1.7	0.006
**PEPD**	3.0	0.007
**PI16**	3.1	0.003
**PIKFYVE**	2.1	0.002
**PLG**	1.5	0.002
**PON1**	3.7	0.01
**PPL**	4.4	0.0001
**PPM1B**	3.3	0.02
**SCF**	1.5	0.0000008
**SELL**	6.7	0.001
**SERPINA6**	1.2	0.004
**SERPINA7**	2.3	0.007
**SERPINC1**	0.8	0.004
**SERPINF2**	4.1	0.0002
**SERPING1**	1.4	0.002
**SERPINI1**	2.1	0.004
**SFRP4**	6.8	0.00001
**SHBG**	4.1	0.007
**SOD3**	3.7	0.006
**TCN1**	2.2	0.01

**TGF-alpha**	3.0	0.006	2.9	0.002
**TNFB**	0.7	0.00002	0.7	0.0000004
**TNFSF14**	3.6	0.00000005	2.2	0.01
**TP53BP1**	2.2	0.0003	1.8	0.03
**uPA**	8.6	0.00000000003	6.2	0.00001

**TF**	0.4	0.0005
**TGFBR1**	4.8	0.0008
**TGOLN2**	2.4	0.007
**TNF**	5.3	0.000006
**TNFRSF17**	1.6	0.01
**TNFRSF9**	2.2	0.00001
**TRAIL**	2.3	0.0001
**TSC1**	2.2	0.003
**TSPYL1**	1.2	0.02
**TTR**	0.9	0.01
**VEGFA**	2.1	0.0004
